# The C-terminal D/E-rich domain of MBD3 is a putative Z-DNA mimic that competes for Zα DNA-binding activity

**DOI:** 10.1093/nar/gky933

**Published:** 2018-10-10

**Authors:** Chi-Hua Lee, Yan-Ping Shih, Meng-Ru Ho, Andrew H-J Wang

**Affiliations:** Institute of Biological Chemistry, Academia Sinica, Taipei 115, Taiwan

## Abstract

The Z-DNA binding domain (Zα), derived from the human RNA editing enzyme ADAR1, can induce and stabilize the Z-DNA conformation. However, the biological function of Zα/Z-DNA remains elusive. Herein, we sought to identify proteins associated with Zα to gain insight into the functional network of Zα/Z-DNA. By pull-down, biophysical and biochemical analyses, we identified a novel Zα-interacting protein, MBD3, and revealed that Zα interacted with its C-terminal acidic region, an aspartate (D)/glutamate (E)-rich domain, with high affinity. The D/E-rich domain of MBD3 may act as a DNA mimic to compete with Z-DNA for binding to Zα. Dimerization of MBD3 via intermolecular interaction of the D/E-rich domain and its N-terminal DNA binding domain, a methyl-CpG-binding domain (MBD), attenuated the high affinity interaction of Zα and the D/E-rich domain. By monitoring the conformation transition of DNA, we found that Zα could compete with the MBD domain for binding to the Z-DNA forming sequence, but not vice versa. Furthermore, co-immunoprecipitation experiments confirmed the interaction of MBD3 and ADAR1 *in vivo*. Our findings suggest that the interplay of Zα and MBD3 may regulate the transition of the DNA conformation between B- and Z-DNA and thereby modulate chromatin accessibility, resulting in alterations in gene expression.

## INTRODUCTION

The interaction between protein and DNA is an important step for various biological processes in living cells. In recent years, it has become apparent that in addition to DNA sequences, which can be recognized by specific proteins, the shape of DNA offers different information to achieve DNA binding specificity (1,2).

The most abundant DNA conformation in cells is B-DNA, a canonical right-handed double helix. In addition, a left-handed Z-DNA occurs transiently during transcription. In 1979, Wang *et al.* first solved the crystal structure of Z-DNA, named for the zig-zag sugar phosphate backbone (3). The Z-DNA formation favors alternating purine/pyrimidine residues and requires more energy than B-DNA (4). *In vitro*, the conformation of Z-DNA can be stabilized by a high salt concentration or chemical modification (e.g., cytosine methylation) (5). *In vivo*, Z-DNA is induced by negative supercoiling generated behind a moving RNA polymerase or by the action of the chromatin remodeling complex (6–8). Moreover, the finding that the Z-DNA binding domain (Zα) is able to induce and stabilize Z-DNA conformation indicates distinct functional roles of Z-DNA in cells. In other words, the formation of Z-DNA is not only a consequence due to DNA torsional stress but also a functional DNA structure regulated by Z-DNA binding proteins, which may cooperate with other proteins that are dependent on the cellular context. Zα was first identified from a human RNA-editing enzyme, adenosine deaminase acting on RNA (ADAR1), and subsequently found in other proteins including DLM1, E3L and PKZ (9–12). These Z-DNA binding proteins are involved in different cellular processes; however, the biological roles of Z-DNA/Z-DNA binding protein have not been fully defined.

Accumulating studies demonstrate that Z-DNA functions in transcriptional regulation by showing that the Z-DNA formation impacts chromatin accessibility. Given that Z-DNA cannot be incorporated into nucleosome, the influence of Z-DNA on nucleosome positioning creates an open chromatin environment that is permissive to the transcriptional machinery necessary for gene expression (13,14). Z-DNA-forming sites are identified in the promoters of many genes and are correlated with reduced nucleosome occupancy, enhanced recruitment of RNA polymerase II and activated gene transcription (15–17). The expression of Z-DNA binding protein in cells can induce and stabilize the Z-DNA formation in the promoter, resulting in gene activation (18). Furthermore, the chromatin remodeling complex, the mammalian SWI/SNF-like BAF complex, activates colony-stimulating factor 1 (CSF1) gene expression by facilitating Z-DNA formation in its promoter, and the deficiency of Z-DNA formation reduces CSF1 gene expression (7,8).

In this study, we chose to identify the proteins associated with Zα to investigate the potential biological function of Zα/Z-DNA. Among the list of identified proteins from the pull-down assay using Zα as the bait protein, we sought proteins involved in transcription. Notably, MBD3 and several components of the nucleosome remodeling and histone deacetylation (NuRD) complex could be pulled down by Zα. MBD3 is one of the methyl-CpG-binding domain (MBD) protein family members and is required for assembly of the NuRD complex (19–21). The MBD3/NuRD complex regulates the expression of many genes involved in diverse biological processes (21–26). For example, MBD3/NuRD-mediated gene silencing is a critical determinant for the pluripotency of embryonic stem cells (ESCs). MBD3 contains a distinct C-terminal acidic tail with aspartate (D) and glutamate (E) repeats; however, the function of the D/E-rich domain of MBD3 is completely unknown. Here, we provide evidence for the high affinity interaction between Zα and the D/E-rich domain of MBD3. In addition, the D/E-rich domain exhibited the ability to mimic DNA and thus could compete with Z-DNA for Zα binding. Dimerization of MBD3 through the interaction of the MBD and the D/E-rich domain attenuated the affinity of Zα and the D/E-rich domain. Furthermore, Zα could compete with the MBD domain for binding to d(CG)_6_ and induce conformational conversion of DNA from B- to Z-DNA. The interplay between Z-DNA binding protein and MBD3 to facilitate the DNA conformational transition between B- and Z-DNA may play a crucial role in gene regulation.

## MATERIALS AND METHODS

### Plasmid construction

The cDNAs of Zα (Zα of human ADAR1; residues 133–209) and MBD3 (full-length human MBD3; residue 1–291) were synthesized (Genscript) with codon optimization for *Escherichia coli* expression. The Zα was amplified by PCR and inserted into EcoRI-XhoI sites of the expression vector pET16b (Novagen), resulting in N-terminal His_10_-tagged protein. The plasmids, pMCSG7-Zα, pMCSG10-MBD3, pMCSG7-MBD3MBD (MBD domain of MBD3; residues 1–72), and pMCSG7-MBDDE (MBD domain of MBD3 connected to the acidic tail; residues 263–291) were constructed by ligation-independent cloning procedure with pMCSG7 or pMCSG10 vector, which produces fusion proteins with N-terminal His_6_-tag and His_6_-GST-tag respectively, followed by a tobacco etch virus (TEV) protease cleavage site (27,28).

### Protein purification

The His_10_-tagged Zα protein was overproduced in *E. coli* BL21(DE3) cells. Bacteria were grown at 37°C in Luria-Bertani (LB) medium and induced with 1 mM isopropyl-β-d-thioglactopyranoside (IPTG) until an optical density of 0.6–0.8 at 600 nm. Cells were harvested after growth for further 3 h at 30°C. A cell pellet was resuspended in the lysis buffer [50 mM Tris–HCl pH 8.0, 300 mM NaCl, 10 mM imidazole, 2 mM TCEP, 1 μg/μl lysozyme (Sigma), 5 U/ml benzonase (Novagen) and EDTA-free protease inhibitor cocktail tablet (Roche)] and incubated for 30 min on ice followed by mechanical disruption by passing the cells through a cell disruptor (Constant Systems Ltd). The lysate was then centrifuged at 25 000 × g for 30 min and the soluble Zα protein was purified by immobilized metal affinity chromatography with a nickel-nitrilotriacetic acid (Ni-NTA) column, followed by gel filtration using a Superdex 75 16/600 column (GE Healthcare).

The constructs, pMCSG7-Zα, pMCSG10-MBD3, pMCSG7-MBD3MBD and pMCSG7-MBDDE were used for purification of tag-free Zα, MBD3, MBD3MBD and MBDDE, respectively. The expression condition of each protein has been optimized to obtain higher levels of soluble protein. For Zα, BL21(DE3) cells were grown and induced using the same procedure as described above; for MBD3, cells were induced with 0.1 mM IPTG for 18 h at 16°C; and for MBD3MBD and MBDDE, with 0.5 mM IPTG for 3 h at 30°C. The soluble proteins were purified by Ni-NTA affinity chromatography using the same procedure as described above. After Ni-NTA purification, TEV was added at an approximate ratio of 1 mg protease per 50 mg of target protein and the reaction mixture was dialyzed overnight at 4°C against a dialysis buffer consisting of 50 mM Tris–HCl pH 7.5, 300 mM NaCl, 1 mM DTT, 5% glycerol. After the cleavage, a reverse purification over the Ni-NTA column was performed to remove the N-terminal tag, uncut protein and protease, followed by gel filtration using a Superdex 75 or a Superdex 200 16/600 column (GE Healthcare).

The purity of each protein was analyzed by SDS-PAGE and mass spectrometry. The protein concentration was estimated by A280 using calculated extinction coefficients.

### Cell culture

Human embryonic kidney 293 (HEK293) cell line was obtained from American Type Culture Collection (ATCC; Manassas, VA) and maintained in Dulbecco's modified Eagle medium (DMEM; 11965; Invitrogen) supplemented with 10% heat-inactivated fetal bovine serum under 5% CO_2_ at 37°C.

### Pull-down assay

HEK293 cells were collected and the pellet was resuspended in the lysis buffer [(50 mM Tris–HCl pH7.5, 1% NP-40, 150 mM NaCl, 10 mM imidazole, 0.1 mM PMSF, 90 U/ml Benzonaes (Novagen) and EDTA-free protease inhibitor cocktail tablet (Roche)]. Lysate was clarified by centrifugation for 30 min at 16 000 × g at 4°C. The protein concentration was measured by using Bradford assay. 20 mg of HEK293 cell lysate was pre-cleaned by adding 1 ml of Ni sepharose (50% slurry) (GE Healthcare) and rotated at 4°C for 1 h. Add 1 mg of purified His_10_-Zα protein (buffer only as a negative control) to the pre-cleaned lysate and rotate at 4°C overnight. To capture His_10_-Zα protein, add 0.2 ml Ni sepharose (50% slurry) and incubate at 4°C for 1 h in a rotation wheel. Beads were then washed by wash buffer with gradually increased salt concentration (50 mM Tris–HCl, pH7.5, 150 mM, 0.5 M and 1 M NaCl; 1 ml for each salt concentration). The washed beads were added elution buffer with gradually increased imidazole concentration (50 mM Tris–HCl, pH7.5, 150 mM NaCl, 100, 200 and 500 mM imidazole; 1 ml for each imidazole concentration). Add trichloroacetic acid to precipitate and concentrate the eluates. The concentrated eluates were resuspended in NuPAGE LDS sample buffer (Invitrogen), run on NuPAGE 12% Bis–Tris gels (Invotrogen) and stained using Instant Blue (Expedeon).

### Liquid chromatography–tandem mass spectrometry (LC–MS/MS) analysis

Samples were prepared by using the standard protocol provided by the Academia Sinica Common Mass Spectrometry Facilities in the institute of Biological Chemistry. In brief, gel pieces were destained in a buffer containing 50% acetonitrile and 25 mM ammonium bicarbonate. Destained samples were reduced with 50 mM dithioerythreitol for 1 h at 37°C, alkylated with 100 mM iodoacetamide at dark for 1 h at room temperature, and digested with trypsin. After digestion, the peptides were desalted by Zip-Tip C18 (Millipore).

To identify Zα-interacting proteins, the entire gel lanes were divided into several horizontal slices and subjected to in-gel trypsin digestion. The digested samples were analyzed by LC–MS/MS using LTQ-Orbitrap XL mass spectrometer (Thermo Fisher Scientific, Bremen, Germany). Raw MS/MS data were converted into peak lists (MGF file) using Raw2MSM (29). The resulting peak lists were searched using Mascot (version 2.5.1; Matrix Science) and the parameters were: database, Swiss-Prot; taxonomy, human; enzyme, trypsin; *m/z*, monoisotopic masses; peptide mass tolerance, 10  ppm; MS/MS ion mass tolerance, 0.6 Da; allowance of up to two missed cleavages; variable modifications, oxidation of methionine (Met) and carbamidomethylation of cysteine (Cys). A protein was considered significant when containing at least two unique peptides of which Mascot scores above the statistically significant threshold (*P* < 0.05).

### Fluorescence polarization (FP) assays

FP experiments were performed in 384-well black plates (3575; Corning) and automated using a Beckman Coulter BioMek 3000 liquid handling system. The final reaction volume was 30 μl in a buffer containing 20 mM HEPES–NaOH pH 7.5, 50 mM NaCl, 1 mM DTT. Samples were measured at an excitation wavelength of 485 nm and an emission wavelength of 535 nm in a Paradigm Multi-Mode Microplate Detection Platform (Molecular Device). The FP values in millipolarization (mP) units were determined by measuring the parallel and perpendicular fluorescence intensity (*I*) and were calculated via the formula: FP value (mP) = 1000 × (*I*_parallel_ − *I*_perpendicular_)/(*I*_parallel_ + *I*_perpendicular_). The data were analyzed using GraphPad Prism 5 (GraphPad Software) and the dissociation constant *K*_d_ and the IC_50_ values were determined by non-linear regression using a one-site total binding model and a dose-response (variable slope) model, respectively. Error bars represent standard deviation from triplicate experiments.

### Oligonucleotides

Purified DNA oligonucleotides, d(CG)_6_ and d(C^m^G)_6_ with or without 6-carboxyfluorescein (6-FAM) conjugated at the 5′ end were purchased from GenScript. To create DNA duplex, a concentrated DNA oligonucleotide was heated to 85°C for 10 min and then slowly cooled to room temperature.

### Peptides

All peptides with or without FITC conjugated at the N-terminus were synthesized by the peptide synthesis facility in the institute of Biological Chemistry, Academia Sinica. The purity of the peptides was determined to be > 95% based on high performance liquid chromatography and mass spectrometry analyses.

### Characterization of complex formation by size exclusion chromatography (SEC)

Equimolar amounts (0.1 mM) of Zα and DE-rich peptide were incubated on ice for 1 h in a buffer containing 20 mM HEPES–NaOH pH 7.5, 50 mM NaCl, 1 mM DTT. The mixture was then loaded on a Superdex 30 16/600 column (GE Healthcare) equilibrated in the same buffer. The fractions from the peaks corresponding to Zα alone and the complex of Zα and DE-rich peptide were collected and used for chemical cross-linking.

To analyze complex formation of Zα and other proteins (MBD3, MBD or MBDDE), the mixtures were incubated on ice for 1 h in a buffer containing 20 mM Tris–HCl pH 7.5, 50 mM NaCl, 1 mM TCEP. The mixture was then loaded on a Superdex 75 or Superdex 200 increase 10/300 column (GE Healthcare).

### Chemical cross-linking

The cross-linking reagents, adipic acid dihydrazide (ADH; Sigma) and 4-(4,6-dimethoxy-1,3,5-triazin-2-yl)-4-methylmorpholinium chloride (DMTMM; Sigma), were used as described previously (30). Before cross-linking, prepare freshly the stock solutions of ADH and DMTMM at concentrations of 100 and 144 mg/ml in 20 mM HEPES–NaOH pH7.5, respectively. The cross-linking reaction was performed by incubating the purified Zα alone or the complex (a final protein concentration of 1 mg/ml) with 8.3 mg/ml ADH and 12 mg/ml DMTMM for 90 min at room temperature. The mixtures were immediately resolved by a NuPAGE 12% Bis–Tris gel (Invotrogen) and stained using Instant Blue (Expedeon).

### Size exclusion chromatography combined with multi-angle light scattering (SEC-MLAS)

SEC−MALS measurements were conducted using a miniDAWN TREOS detector and an Optilab T-rEX differential refractive index detector (Wyatt Technology Corporation) coupled to an Agilent 1260 Infinity HPLC system. The SEC experiments were performed on an ENrich SEC 70 10 × 300 column (Bio-Rad) equilibrated in a buffer containing 20 mM HEPES–NaOH pH 7.5, 50 mM NaCl, 1 mM TCEP and 0.02% NaN3. The protein Zα alone (2.7 mg/ml) or the mixture of Zα and the D/E-rich peptide at various molar ratios and incubation periods as indicated was loaded onto the column at a flow rate of 0.5 ml/min. Bovine serum albumin (A1900; Sigma) was used for system calibration and molecular weights were calculated using ASTRA 6 software (Wyatt Technology Corporation) with a d*n*/d*c* value of 0.185 ml/g.

### Circular dichroism (CD)

CD spectra were measured at 24°C on a J-815 Spectrometer (JASCO) using a 1 mm path length quartz cell. Prepare 7.5 μM DNA duplex in a buffer consisting of 5 mM HEPES–NaOH pH 7.5, 25 mM NaCl and 1 mM DTT, and in the absence or presence of indicated concentration of proteins. Spectra were recorded between 235 and 305 nm wavelength with a data pitch of 1 nm, bandwidth of 1 nm and with two accumulations at a scanning speed of 10 nm/min.

### Bioinformatics analysis of identified proteins

Zα-interacting protein list from LC–MSMS was analyzed in UniProt (31) (http://www.uniprot.org) to search the biological processes in which the proteins are involved. To find out proteins participate in transcription, UniProt keywords ‘transcription’ were used. Association network of proteins were constructed by STRING (version 10.5) (32) (https://string-db.org). Each connection was derived from experimental evidence and with a minimum score of 0.7 (high confidence). Hide disconnected nodes in the network. The resulting association network was visualized by Cytoscape (version 3.0.0) (33).

### Transient transfection

For expressing FLAG-MBD3 and its mutants in 293F cells (Freestyle™ 293 Expression System; Invitrogen), the DNA fragments were PCR amplified and inserted into HindIII site of pFLAG-CMV-2 vector (Sigma-Aldrich). The plasmids were purified by EasyPrep EndoFree Maxi Plasmid Extraction Kit (BIOTOOLS Co., Ltd, Taiwan) and transiently transfected into 293F cells using 293fectin (Invitrogen) according to the manufacturer's guidelines.

### Immunoprecipitation and immunoblotting

After 48 h posttransfection, the cells were washed with cold PBS and lysed with lysis buffer [50 mM Tris–HCl pH 7.5, 0.3% NP-40, 100 mM potassium acetate, 10% glycerol, 50 U/ml Benzonaes (Novagen) and EDTA-free protease inhibitor cocktail tablet (Roche)]. The lysates were incubated with anti-FLAG M2 Affinity Gel (A2220; Sigma-Aldrich) on a rotation at 4°C overnight. The resin was then washed three times with wash buffer I (50 mM Tris–HCl pH7.5, 100 mM potassium acetate, 5% glycerol) and three times with wash buffer II (50 mM Tris–HCl pH 7.5, 150 mM NaCl). The immunoprecipitates were eluted by FLAG peptide (F4799; Sigma-Aldrich) and resolved by a NuPAGE 12% Bis–Tris gel (Invotrogen). Specific antibodies used for western blotting were Rabbit anti-FLAG (1:5000; F7425; Sigma-Aldrich) and rabbit anti-ADAR1 D7E2M (1:1000; 14175; Cell Signaling Technology).

## RESULTS

### Affinity purification and mass-spectrometric identification of Zα-interacting proteins

**Figure 1.**
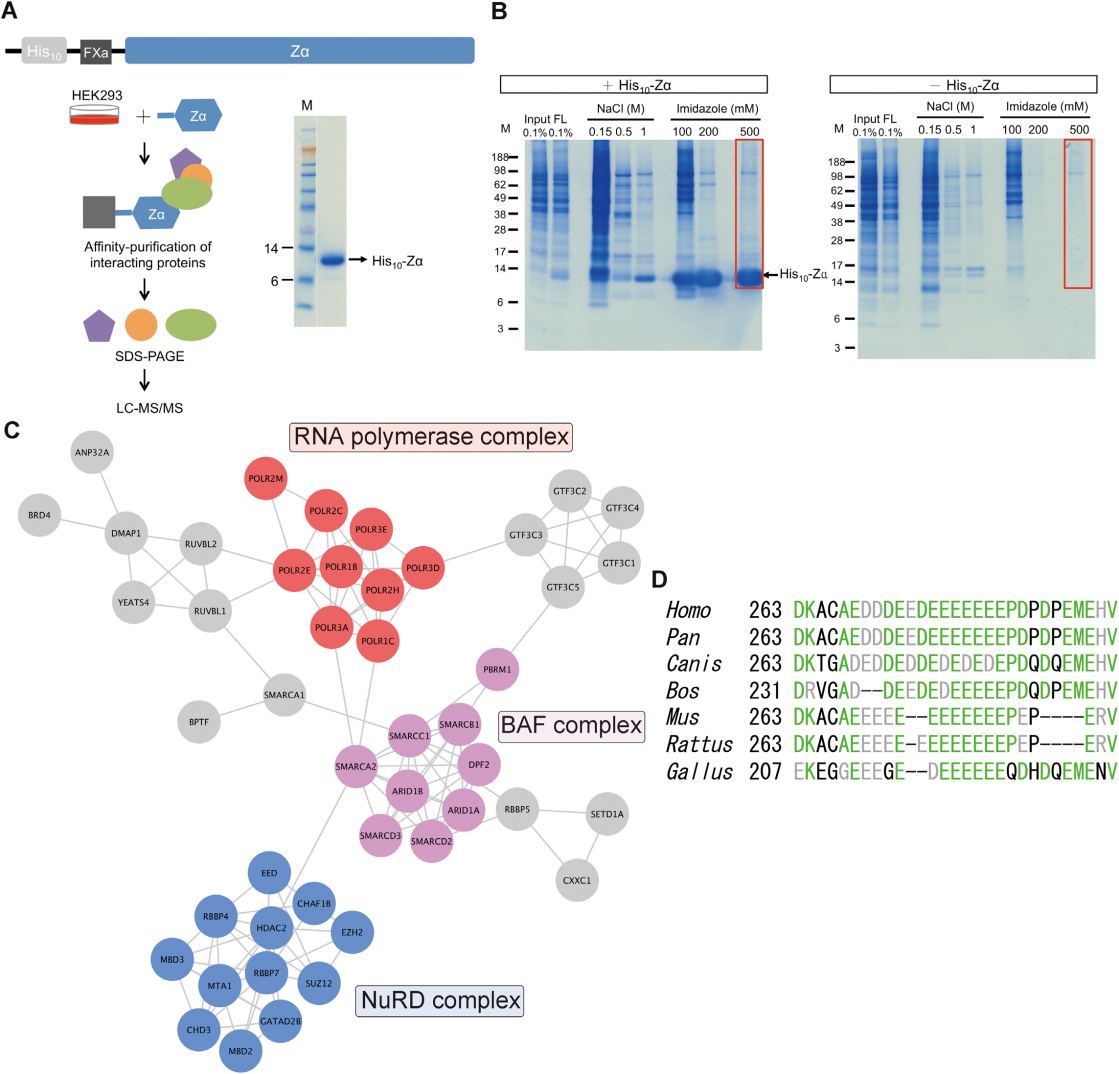
Identification of proteins associated with His_10_-Zα protein. (**A**) Schematic representation of His_10_-Zα protein and mass spectrometric identification of Zα-interacting proteins. The purity of His_10_-Zα protein was observed by SDS-PAGE with Coomassie blue staining. FXa, FactorXa cleavage site. (**B**) SDS-PAGE analysis of Zα-interacting proteins from the pull-down assay. His_10_-Zα protein or negative control (buffer only) was incubated with HEK293 cell lysates and then affinity-purified using Ni-agarose beads. The bead-associated proteins were eluted and separated by SDS-PAGE with subsequent Coomassie blue staining. The final eluates shown in the red boxes were analyzed by LC–MS/MS. M, protein markers. FT, flow through. (**C**) STRING network analysis of Zα-interacting protein candidates that participate in transcription. (**D**) Alignment of the D/E-rich tail of MBD3 homologs. Multiple sequence alignment was performed by using M-Coffee and the resulting alignment was processed by BoxShade. Identical and similar amino acids are shaded green and grey, respectively. Seven protein sequences of MBD3 homologs were used including human (Homo sapiens, NP_001268382.1), chimpanzee (*Pan troglodytes*, XP_512241.1), gray wolf (*Canis lupus*, XP_868594.1), cow (*Bos taurus*, NP_001121977.1), mouse (*Mus musculus*, NP_038623.1), brown rat (*Rattus norvegicus*, NP_001102205.1), chicken (*Gallus gallus*, NP_001073219.1).

To understand the biological function of Zα/Z-DNA, we sought to identify Zα interacting proteins using a pull-down approach combined with liquid chromatography-tandem mass spectrometry (LC–MS/MS; Figure 1A). In brief, HEK293 cell lysates were incubated with recombinant His_10_-tagged Zα protein and then Zα-interacting proteins were co-purified using Ni-agarose beads and separated by SDS-PAGE (Figure 1B). After very stringent wash and elution steps, there were relatively few proteins with nonspecific binding to the Ni-agarose beads (Figure 1B; compare the last lanes of the two gels). Since low abundance proteins may not be clearly seen in the stained gel, for sensitive proteomic analysis, we divided the lane into several horizontal slices and digested each of them with trypsin for protein identification. The tryptic peptide samples were analyzed individually by LC–MS/MS. To assess the extent of nonspecific binding, we also analyzed the eluates from the beads of the control pull-down. In total, 837 Zα-interacting protein candidates were identified after removing the proteins present in the control sample. The identified proteins were involved in diverse biological processes, including transport, transcription, mRNA processing and cell cycle, among others, according to the analysis in UniProt (31) (http://www.uniprot.org).

Since we aimed to study the function of the interaction between Zα and Z-DNA, we focused on the 122 proteins that participate in transcription and analyzed the association network of those proteins using the STRING database (32) (https://string-db.org). Interestingly, we found that these proteins could be grouped functionally into three multimeric protein complexes: RNA polymerase, BAF, and NuRD complex (Figure 1C). The association of Zα with RNA polymerase or the BAF complex might be expected and correlated to the function of Zα, since it is known that the negative torsional strain generated by the moving RNA polymerase during transcription and the chromatin remodeling activity of BAF complexes can cause the Z-DNA formation (6–8). The addition of Zα can further stabilize the Z-DNA conformation, in comparison with Z-DNA *per se*, to significantly increase gene expression levels (18). Therefore, a Zα-containing protein may be recruited by RNA polymerase or the BAF complex to maintain the Z-DNA conformation in promoter regions, and the open conformation allows entry of transcription factors (4,7,13), resulting in increased expression of genes.

Unexpectedly, the NuRD complex was pulled down by Zα. Until now, there is no evidence showing the relationship between the NuRD complex with Zα/Z-DNA. However, we noticed that the core component of the NuRD complex, MBD3, contained a particular D/E-rich domain in the C-terminus. According to our previous studies investigating D/E-rich repeat structures and DNA mimic proteins, those D/E residues may mimic the phosphate backbone of DNA/RNA (34,35). Thus, the D/E-rich domain of MBD3 has the potential to interact with the Z-DNA binding pocket of Zα. Multiple sequence alignment of MBD3 proteins has revealed that the characteristics of the D/E-rich domain are conserved in many species, although the length and composition of the acidic tails are not identical (Figure 1D). For example, the core acidic regions of mouse and rat MBD3 simply contain glutamic acid residues, but not a combination of aspartic acid and glutamic acid residues like the others.

### Zα interacts with the C-terminal D/E-rich domain of MBD3

**Figure 2.**
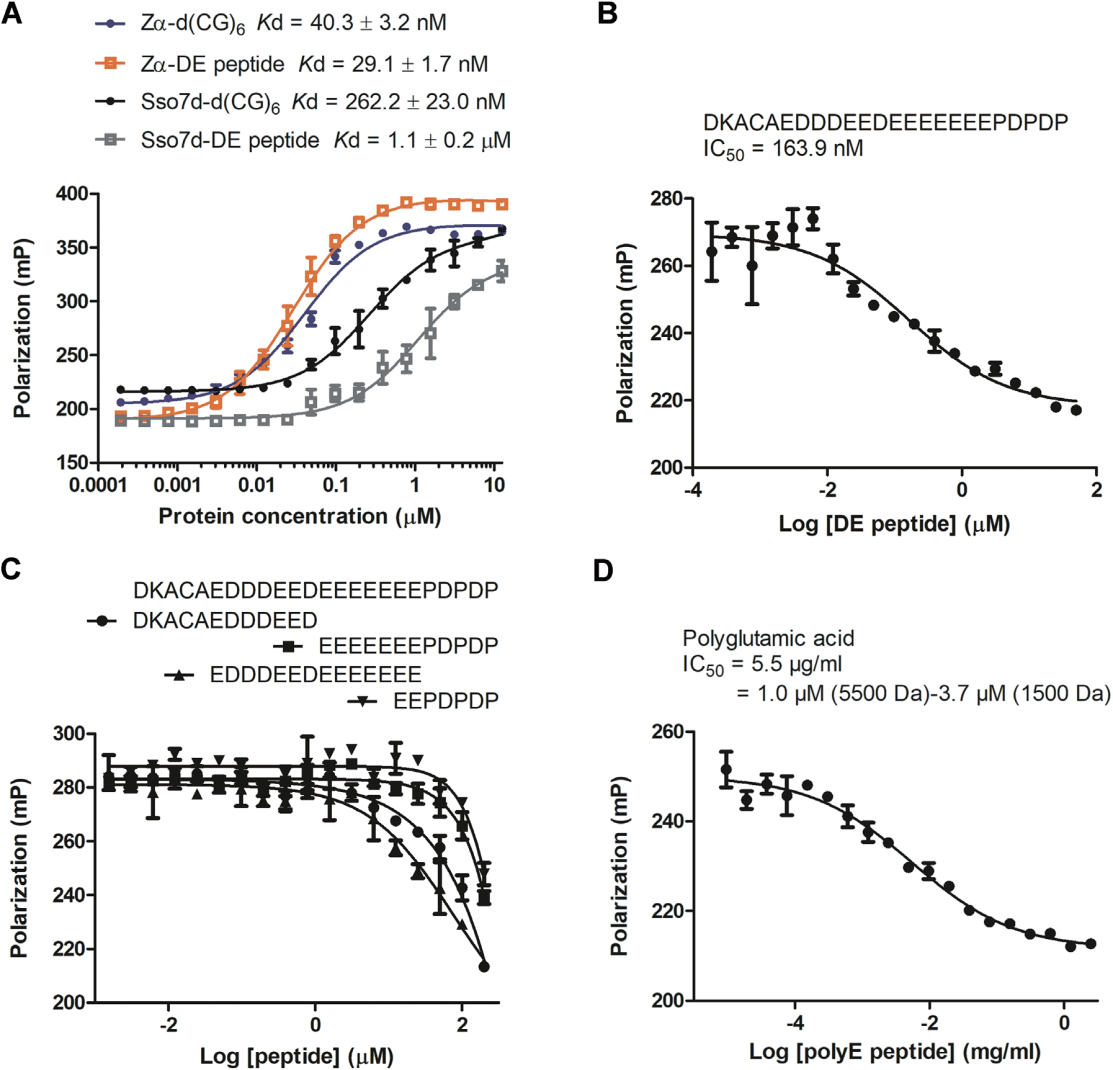
Zα interacts with the D/E-rich domain of MBD3. (**A**) FP assay to obtain *K*_d_ using fluorescently labeled d(CG)_6_ duplex [6-FAM-d(CG)_6_] or D/E-rich peptide (FITC-DE) against Zα or Sso7d. Competitive binding curve of the pre-incubated mixture of Zα and 6-FAM-d(CG)_6_ duplex against (**B**) full-length, (**C**) truncated D/E-rich peptides or (**D**) poly-L-glutamic acid (1500–5500 Da; Sigma) peptide. The error bars in each plot represent the mean ± standard deviation of triplicate experiments. mP, millipolarization.

We then performed a fluorescence polarization (FP) assay to examine the affinity between Zα and the D/E-rich domain of MBD3 (Figure 2A). The results showed that Zα bound to the D/E-rich peptide (*K*_d_ = 29.1 ± 1.7 nM) with high affinity similar to Zα binding to a CG repeat DNA, d(CG)_6_ (*K*_d_ = 40.3 ± 3.2 nM). However, a general DNA binding protein, Sso7d (36), bound to the D/E-rich peptide with lower affinity (*K*_d_ = 1.1 ± 0.2 μM). It is noteworthy that in contrast to Zα, the binding affinity of Sso7d to the D/E-rich peptide was approximately 4-fold lower than Sso7d binding to d(CG)_6_ (*K*_d_ = 262.2 ± 23.0 nM) although both Zα and Sso7d contain positively charged DNA-binding surfaces that complement the negatively charged DNA. Next, we investigated whether the D/E-rich peptide could compete with Zα for binding to d(CG)_6_. Unlabeled D/E-rich peptide displaced Zα binding to d(CG)_6_ in a concentration-dependent manner, resulting in 50% displacement at 163.9 nM (95% confidence interval (CI), 72.9–368.7 nM; Figure 2B). Interestingly, only full-length but not truncated D/E-rich sequence could compete with d(CG)_6_ for binding to Zα (Figure 2C). Furthermore, the polymeric synthetic acidic peptide, polyE, could compete with Zα binding to d(CG)_6_, although the efficiency was lower than full-length D/E-rich peptide (Figure 2D). The concentration at 50% displacement was 5.5 μg/ml (between 1.0 and 3.7 μM for a mixture of polyE with a molecular weight of 1500–5500 Da; 95% CI, 3.4–8.9 μg/ml). Taken together, the results indicate that Zα specifically interacts with the D/E-rich domain of MBD3.

### Zα forms complexes with the D/E-rich domain

**Figure 3.**
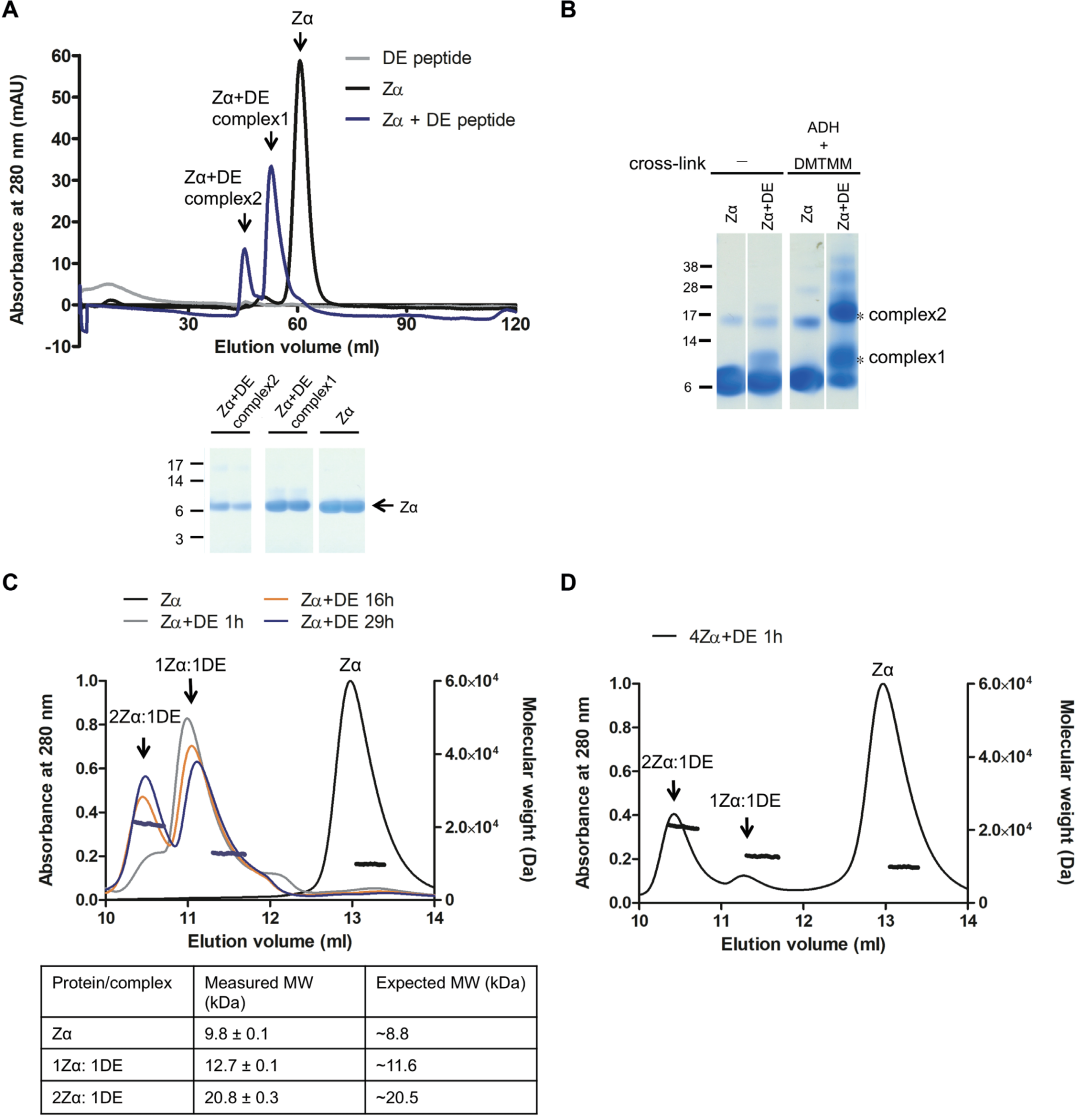
Analysis of complex formation of Zα and the D/E-rich domain. (**A**) Size-exclusion chromatography profiles of Zα (black), D/E-rich peptide (grey) and an equimolar mixture of Zα and D/E-rich peptide on a Superdex 30 16/600 column. The bottom panel shows SDS-PAGE analysis of fractions from peaks. mAU, milliabsorbance unit. (**B**) SDS-PAGE analysis of the Zα-DE complex cross-linked with ADH and DMTMM, which target Asp and Glu residues. (**C**) SEC-MALS analysis of Zα alone (black) and equimolar mixtures of Zα and D/E-rich peptide after incubation for 1 (gray), 16 (orange) and 29 h (blue). (**D**) The SEC-MALS profile of the mixture of Zα and D/E-rich peptide, which were combined at a 4:1 molar ratio and incubated on ice for 1 h. The left *y* axis represents the normalized UV absorbance at 280 nm, while the right *y* axis represents the molecular weight (Da). The horizontal traces show the calculated molecular weight. DE, full-length of D/E-rich peptide.

To examine the complex assembly of Zα and the D/E-rich domain, we conducted size exclusion chromatography (SEC) and then chemical cross-linking. The SEC experiment showed that two shifted peaks, which represented two types of complexes, formed after incubation of Zα and the D/E-rich peptide (Figure 3A). We collected the elution fractions from the shifted peaks and confirmed the presence of Zα in each peak by SDS-PAGE analysis (Figure 3A). The complexes were then subjected to cross-linking. Since the peptide contained a large number of Asp and Glu, the lysine (Lys) specific cross-linking reagent was not favorable for the complex. To cross-link side-chains of Asp and Glu, we used the cross linker adipic acid dihydrazide (ADH) and the coupling reagent 4-(4,6-dimethoxy-1,3,5-triazin-2-yl)-4-methylmorpholinium chloride (DMTMM), which can couple carboxyl groups with hydrazides at neutral pH (30). Previously, Leitner *et al.* demonstrated that the maximal calculated Cα distance of linkable Asp and Glu residues is 21 Å. In addition, Lys and acidic residues can form zero-length cross-links, and the cut-off value may be ∼25 Å. After the cross-linking reaction, the samples were immediately analyzed by SDS-PAGE and two higher molecular weight species were observed between 10 and 22 kDa (Figure 3B). To obtain the precise MW of the complexes, we performed size-exclusion chromatography coupled with multi-angle light scattering (SEC-MALS) analysis. The data showed that the measured MWs of the two shifted peaks were 20.8 and 12.7 kDa, in agreement with the theoretical calculated MW of 2:1 (20.5 kDa) and 1:1 (11.6 kDa) stoichiometries of Zα-D/E-rich peptide complexes, respectively (Figure 3C). In addition, when an equimolar concentration of Zα and D/E-rich peptide was incubated for 1 h, the 1:1 complex was predominantly formed, and the amount of 2:1 complex was relatively low. The amount of the 2:1 complex increased with an increasing incubation time. The time-dependent change was accelerated by addition a 4-fold excess amount of Zα. After incubation for 1 h, the 2:1 complex was predominantly formed, and free Zα could be detected (Figure 3D). These data confirm that the D/E-rich domain of MBD3 is sufficient for high-affinity binding to Zα and that one D/E-rich domain can bind to two Zα.

### Dimerization of MBD3 inhibits the interaction between Zα and the D/E-rich domain

By using the short fragment, the D/E-rich peptide, we demonstrated the high-affinity interaction and detected complex formation between Zα and the D/E-rich domain of MBD3. To further examine whether Zα could form a stable complex with full-length MBD3 protein by virtue of its interaction with the D/E-rich domain, we constructed full-length human MBD3 in the bacterial expression vector for recombinant protein purification. The *Escherichia coli*-expressed MBD3 protein was unstable with a large amount of aggregation and degradation. Presumably, the instability might be partly due to the long disordered region, especially the D/E-rich domain in the C-terminus, since amino acid compositional bias is a common feature of disordered sequence (Supplemental Figure S1) (37). The disordered region thus increases the susceptibility to proteolytic degradation of MBD3 protein.

**Figure 4.**
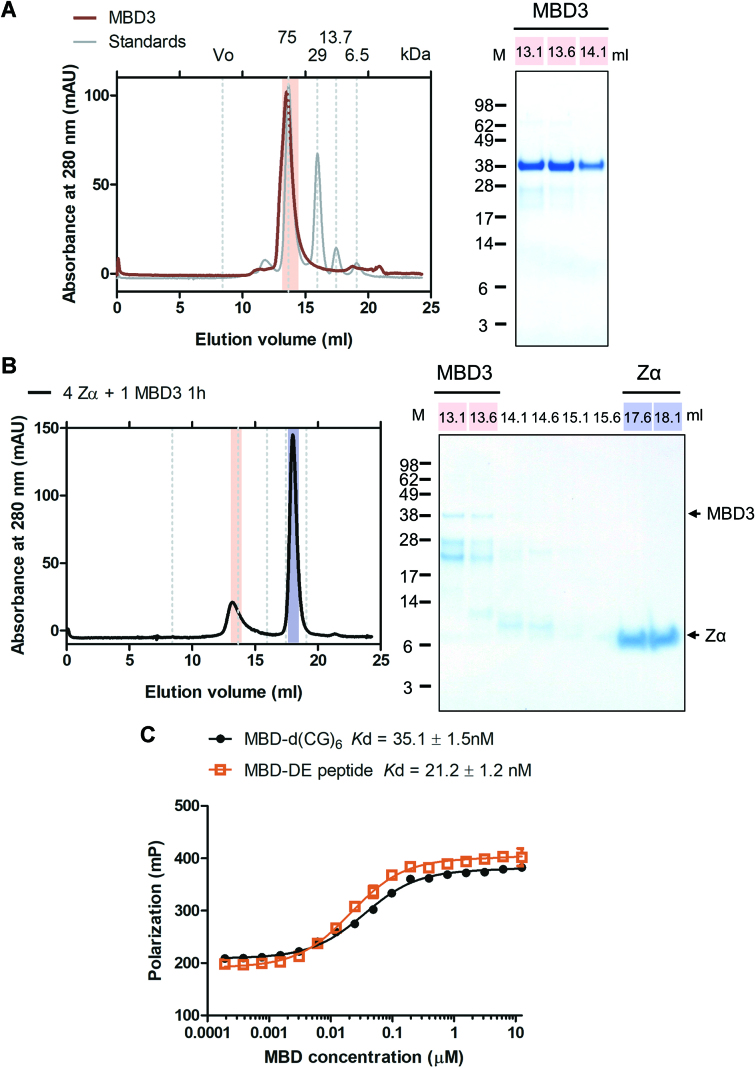
Dimerization of MBD3 inhibits the interaction between Zα and the D/E-rich domain. (**A**) The SEC profile of purified human MBD3 (red) and protein standards (gray) on a Superdex 200 increase 10/300 GL column. The elution positions of protein standards are indicated by grey dotted lines and their MWs are shown on the top. MBD3 elutes at a position corresponding to a dimer (the theoretical MW of the monomer is approximately 33.1 kDa). The peak was analyzed by SDS-PAGE with Coomassie blue staining. (**B**) SEC of the mixture of Zα (100 μM) and MBD3 (25 μM) at a 4:1 molar ratio on a Superdex 200 increase 10/300 GL column. The fractions were analyzed by SDS-PAGE with Coomassie blue staining. mAU, milliabsorbance unit. (**C**) FP assay to obtain *K*_d_ using 6-FAM-d(CG)_6_ or FITC-DE against the MBD domain.

The soluble MBD3 protein appeared to form a dimer in gel filtration by comparing the elution volume to those of standard markers (Figure 4A). Previous experiments have shown that increasing the amount of Zα accelerated the rate of complex formation. Hence, the two proteins, Zα and MBD3, were incubated at a ratio of 4:1 and then subjected to SEC followed by SDS-PAGE analysis of the eluted fractions. However, there were almost no detectable stable complex formation of Zα and MBD3 (Figure 4B).

**Figure 5.**
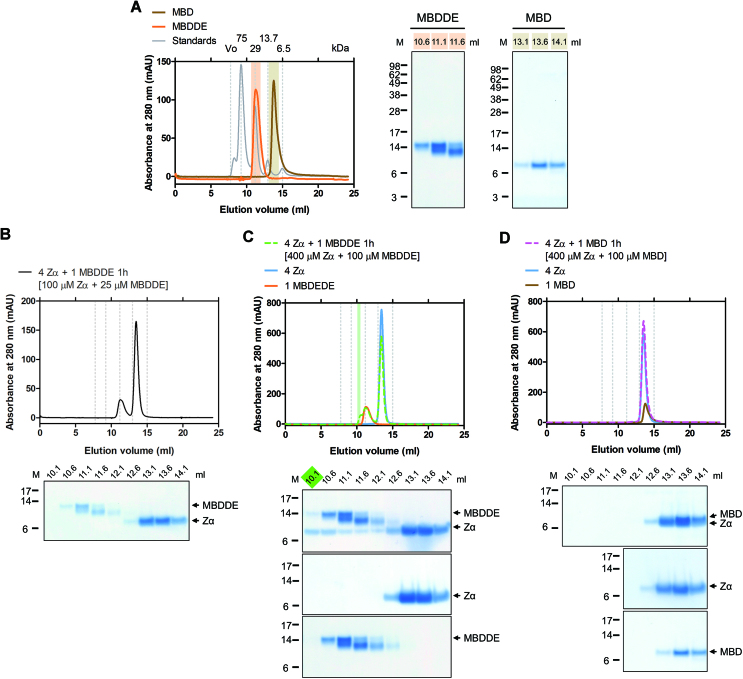
Dimerization of MBDDE inhibits the interaction between Zα and the D/E-rich domain. (**A**) The SEC profile of purified MBDDE (orange), MBD (brown) and protein standards (grey) on a Superdex 75 10/300 GL column. Elution positions of protein standards are indicated by grey dotted lines, and their MWs are shown at the top. MBDDE elutes at a position corresponding to a dimer (the theoretical MW of the monomer is approximately 12 kDa), while MBD is monomer (the theoretical MW of the monomer is approximately 8.6 kDa). The peak was analyzed by SDS-PAGE with Coomassie blue staining. (**B**, **C**) SEC of the mixture of Zα and MBDDE at a 4:1 molar ratio on a Superdex 75 10/300 GL column. The concentration of each protein used is shown at the top. (**D**) SEC of the mixture of Zα and MBD at a 4:1 molar ratio on a Superdex 75 10/300 GL column. The fractions were analyzed by SDS-PAGE with Coomassie blue staining. mAU, milliabsorbance unit.

We noticed that MBD3 contained an N-terminal DNA-binding domain, methyl-CpG binding domain (MBD) although the selective binding affinity of the MBD domain for CpG or modified CpG, 5-methylacytosine (mCpG) and 5-hydroxymethylcytosine (hCpG), has remained controversial (24,38–40). Since the D/E-rich peptide was able to bind DNA-binding proteins (Figure 2A), the C-terminal D/E-rich domain of MBD3 might associate with the N-terminal MBD domain *in trans* to form a homodimer. The intermolecular interaction thus blocked both interfaces of the MBD domain for DNA binding and the D/E-rich domain for Zα binding. The FP assay results indeed showed that the MBD domain interacted with the D/E-rich peptide with high affinity similar to MBD binding to DNA (Figure 4C). Accordingly, Zα and the MBD domain exhibited a similar affinity to the D/E-rich peptide. Zα was supposed to interact with MBD3 by competing with the intermolecular interaction between the MBD and the D/E-rich domain, but less efficiently than the interaction between Zα and the D/E-rich peptide. Therefore, the undetected complex formation of Zα and MBD3 might be caused by a less efficient reaction, resulting in amounts that are too low to be detected. To provide further evidence for the competition mechanism, we created a truncated construct containing only the MBD domain connected to the D/E-rich tail (hereafter referred to as ‘MBDDE’). The *E. coli*-expressed MBDDE protein was more sensitive to protease than the MBD domain, and degradation mainly occurred from the C-terminal disordered region according to the mass spectrometry analysis (Figure 5A). Interestingly, the MBDDE protein eluted much earlier than the MBD domain during gel filtration, despite the small difference in MW between the two proteins (∼3.4 kDa). Based on their elution positions, the MBDDE protein was present in the peak corresponding to its dimeric form, while the MBD domain was a monomer (Figure 5A). The MBDDE protein formed a homodimer through the intermolecular interaction between the MBD and the D/E-rich domain.

We then subjected the MBDDE protein to SEC experiments in the presence of a 4-fold molar excess of Zα. Again, we could not detect stable complex formation of Zα and MBDDE by using lower amounts of proteins (Figure 5B). However, when we raised the concentrations of MBDDE (from 25 to 100 μM) and Zα (from 100 to 400 μM), a small amount of MBDDE and Zα complex was detected by a shift in the SEC profile, and the co-elution of the two proteins was analyzed by SDS-PAGE (Figure 5C). Significantly, Zα interacted with MBDDE less efficiently than with D/E peptide, indicating that the D/E-rich domain was blocked by the MBD domain for Zα binding. The dimerization of MBDDE appeared to restrict the access of Zα to the D/E-rich domain. Addition of an excess amount of Zα could compete with MBD for binding to the D/E-rich domain, leading to disruption of the homodimer of MBDDE and complex formation of Zα and MBDDE. The SEC and SDS-PAGE analysis displayed a mixture in solution that included the homodimer of MBDDE and 1:1 and 2:1 complexes of Zα and MBDDE, despite the fraction (10.1 ml; highlighted in green in Figure 5C) from the shifted peak containing only the 2:1 complex of Zα and MBDDE. We also examined the interaction between the MBD domain and Zα as a control, while no complex formation was detected under the same conditions (Figure 5D).

### Zα and the MBD domain compete for binding to the Z-DNA-forming sequence

**Figure 6.**
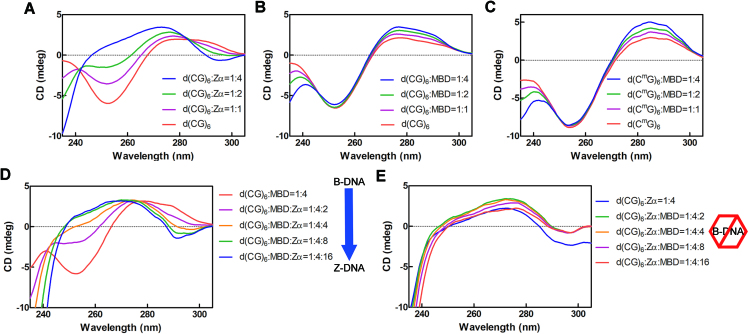
Zα can compete with the MBD domain for binding to d(CG)_6_, but not vice versa. (**A**) CD spectra of the d(CG)_6_ duplex in the presence of Zα. CD spectra of (**B**) the d(CG)_6_ and (**C**) the d(C^m^G)_6_ duplex, respectively, in the presence of the MBD domain. (**D**) Monitoring of the change in CD spectra with the addition of increased amounts of Zα to the pre-incubated mixture of the MBD domain and d(CG)_6_. (**E**) Monitoring of the change in CD spectra with the addition of increased amount of the MBD domain to the pre-incubated mixture of Zα and d(CG)_6_. Spectra are expressed in absolute values of ellipticity in millidegrees (mdeg).

Previous FP data have revealed that the MBD domain binds to the Z-DNA-forming sequence, d(CG)_6_ with a similar high affinity as Zα. However, the patterns of protein-DNA interactions for the MBD domain and Zα are very different. The structure of the MBD domain contains a four-stranded β-sheet followed by a single α-helix and a C-terminal loop, and it contacts the major groove of DNA by using the central two strands of the β-sheet to form a long finger-like projection (39). In contrast, Zα, with the winged helix-turn-helix folding topology, contacts only one strand of the DNA duplex, and the interface is achieved through direct or water-mediated interactions between protein side chains and the sugar-phosphate backbone of Z-DNA (41). The interaction between Zα and Z-DNA is in a conformation-specific manner. Thus, the MBD domain is presumably able to bind to d(CG)_6_ and maintain it in the B-DNA conformation, but it is unable to convert it to Z-DNA. We conducted circular dichroism (CD) experiments to examine the change in the DNA conformation after it was bound by Zα or the MBD domain. Indeed, the MBD domain kept the duplex DNA in B-form showing a positive band at approximately 295 nm and a negative band around 255 nm, while Zα induced the Z-DNA formation, showing inverse CD signals at approximately 255 nm and 295 nm (Figure 6A–C). Therefore, the CD spectrum was distinguishable when the MBD domain or Zα bound to the duplex DNA. Taking advantage of this property, we further examined whether Zα and the MBD domain could compete with each other for binding to d(CG)_6_ by monitoring the DNA conformational change. With the addition of an increased amount of Zα into the pre-incubated mixture of the MBD domain and d(CG)_6_, we observed that the conformation of the duplex DNA changed from B- to Z-from. The transition indicate that Zα is able to compete with the MBD domain for binding to d(CG)_6_ (Figure 6D).

Conversely, Zα protein was pre-incubated with d(CG)_6_, but the conformation of DNA was not changed with the addition of an increasing amount of the MBD domain, indicating that the MBD domain could not compete with the binding of Zα and Z-DNA (Figure 6E). In conclusion, Zα can compete with the MBD domain for binding to d(CG)_6_, but not vice versa.

### MBD3 interacts with ADAR1 *in vivo*

Human cells contain two isoforms of ADAR1, p150 and p110, due to alternative promoter usage and alternative splicing (42). P150 contains two Z-DNA binding domains (Zα and Zβ), three RNA binding motifs and a catalytic editing domain, whereas the p110 isoform lacks Zα but contains all the other domains.

**Figure 7.**
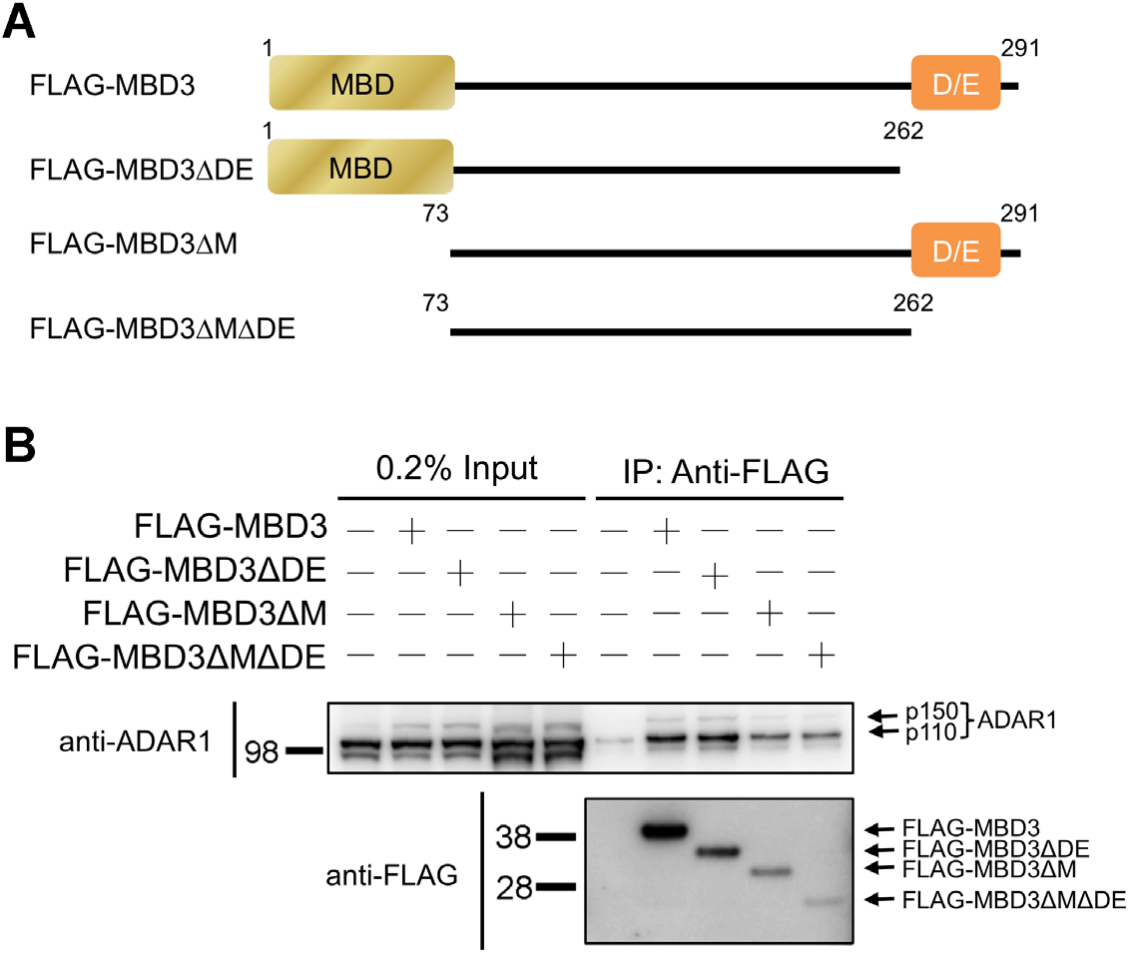
MBD3 interact with ADAR1 *in vivo*. (**A**) The domain structures of MBD3 and its truncation mutants are shown. (**B**) The full-length FLAG-MBD3 and its truncation mutants were expressed in 293F cells, and whole-cell extracts were subjected to FLAG-IP, followed by immunoblotting with the indicated antibodies.

The above data have demonstrated that the DE-rich domain of MBD3 is sufficient for the association with Zα *in vitro*. To examine whether MBD3 is able to interact with endogenous ADAR1 in cells, we transiently expressed FLAG-tagged MBD3 in 293F cells and performed an immunoprecipitation (IP) experiment with anti-FLAG resin. Endogenous ADAR1 was detected by western blotting using anti-ADAR1 antibody. The results showed that endogenous ADAR1 was indeed pulled down by FLAG-MBD3, demonstrating that MBD3 could interact with ADAR1 *in vivo* (Figure 7B). Our *in vitro* experiments have indicated that dimerization of MBD3 inhibits the interaction between Zα and the D/E-rich domain. Thus, to prevent the inhibitory effect caused by dimerization and to determine whether the DE-rich domain of MBD3 was required for the association with ADAR1, we created various FLAG-MBD3 variants with a truncation of the DE-rich domain, the MBD domain or both domains (Figure 7A). The cell extracts with overexpression of FLAG-MBD3 truncation mutants were used for IP experiments. The results showed that endogenous ADAR1 was immunoprecipitated by either full-length MBD3 or its variants (Figure 7B), indicating that the interaction of MBD3 and ADAR1 occurred not only through the interaction of the DE-rich domain and Zα but also other regions.

## DISCUSSION

**Figure 8.**
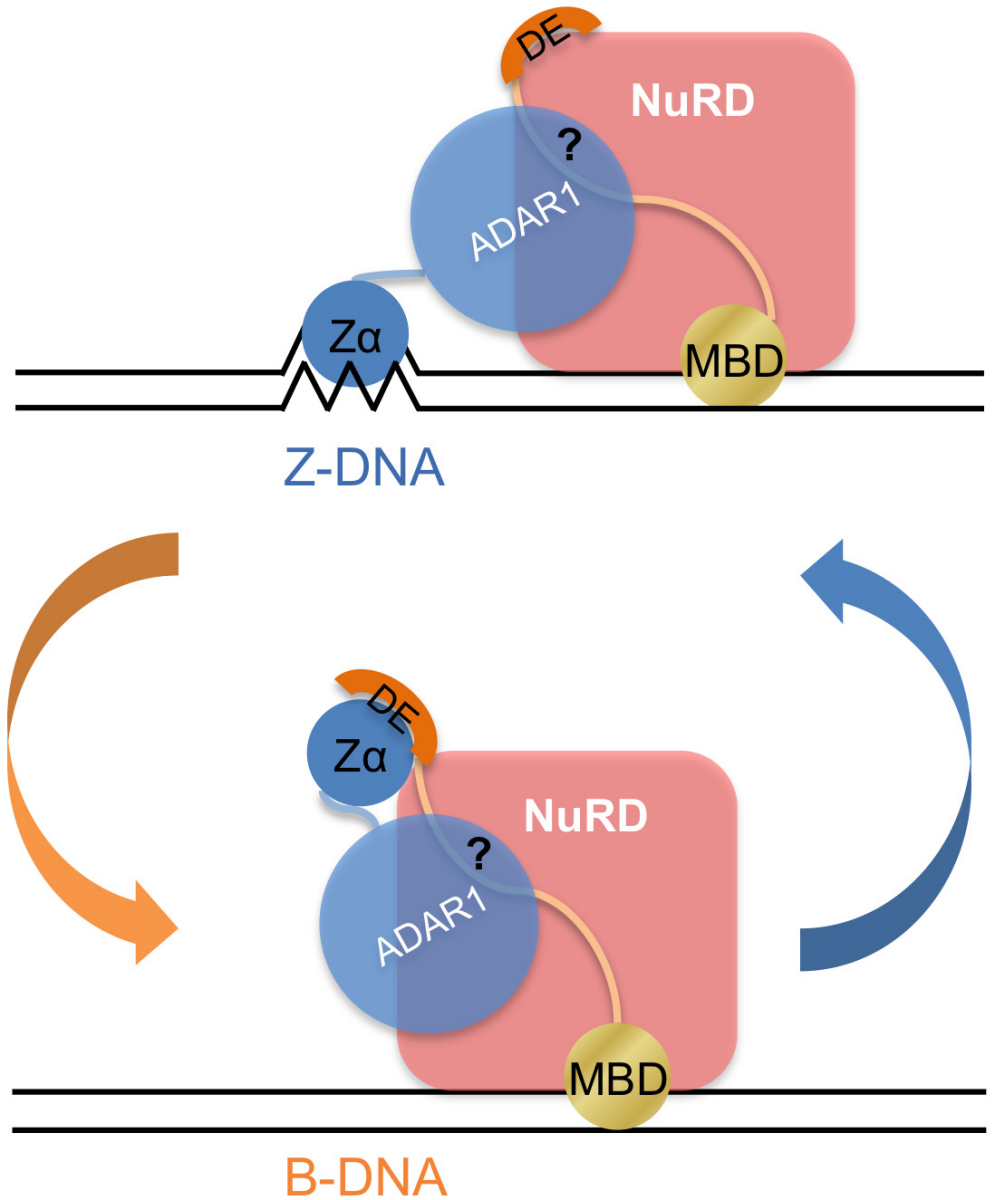
Schematic representation of the proposed model shows that the interplay of Zα and MBD3 modulates the transition of the DNA conformation.

The ability of Zα to induce and maintain the Z-DNA conformation led us to explore the protein interaction network of Zα, which is correlated with the function of Zα/Z-DNA. Using a pull-down assay, we identified a novel Zα-interacting protein, MBD3, and demonstrated that the D/E-rich domain of MBD3 was sufficient for the Zα interaction. The D/E-rich domain might act as a DNA mimic to compete with Z-DNA for binding to Zα. Dimerization of MBD3 via the interaction of the MBD and the D/E-rich domain attenuated the high binding affinity of Zα to the D/E-rich domain. Furthermore, we demonstrated that Zα could compete with the MBD domain for binding to d(CG)_6_ and induce conformational conversion of DNA from B- to Z-DNA, but not vice versa. Taken together, our findings lead to a model in which Zα and MBD3 participate in regulating the transition of the DNA conformation (Figure 8).

### The D/E-rich domain of MBD3 acts as a putative DNA mimic

This is the first report to identify a particular protein sequence with D/E repeats capable of competition with Z-DNA for binding to Zα, while the potential of acidic residues for DNA mimicry, referring to B-DNA or ssDNA, have been observed previously. The solution structure of the *Drosophila* TBP-TAFII230 complex shows that the distribution of the negatively charged side chains of TAFII230 mimic the positions of phosphate groups of the TATA element (43). Our previous studies in relation to D/E-rich repeat structures and DNA mimic proteins also suggest that D/E residues mimic the phosphate backbone of DNA/RNA (34,35). Additionally, the number of D/E residues and the surrounding non-D/E residues may contribute to the binding specificity. The D/E-rich segment (EDWAGLDEDED) of DSS1 acts as a DNA mimic to attenuate the affinity of replication protein A (RPA) for ssDNA (44). Mutation of these solvent-exposed acidic residues and tryptophan to alanine strongly impairs the ability of DSS1 to interact with RPA. Mutants with fewer changes in those residues are less impaired for RPA association. Moreover, to create DNA-mimicking peptides for practical usage, the features of DNA mimic proteins/peptides, the size of the peptides and the positions of those acidic residues in three-dimensional space should be considered (45). Our results herein are in agreement with those findings. The D/E-rich peptide used in this study not only contains D/E residues but also alanine, cysteine, lysine and proline. Only full-length but not truncated D/E-rich sequence can compete with d(CG)_6_ for binding to Zα, which can be performed by simple E repeats with less efficiency. Interestingly, the D/E-rich peptide also shows high affinity for the MBD domain, a B-DNA binding protein, indicating that the D/E-rich peptide with inherent flexibility is able to adopt different conformations when binding to Zα or the MBD domain. However, due to the lack of structural analysis in this study as well as a few D/E repeat structures that to date are known in the Protein Data Bank, the relative contribution of these residues to the binding properties remains unknown and must be further addressed.

### The homodimeric form of MBD3 inhibits Zα binding to the D/E-rich domain

MBD3 was identified as a member of the MBD family approximately two decades ago (19). Many studies have demonstrated that MBD3, an indispensable component of the NuRD complex, plays a critical role in transcriptional regulation (for reviews, see (46,47)). Most of those studies have focused on the function of the MBD domain. Although the D/E-rich sequences of MBD3 orthologs are conserved in vertebrates (Figure 1D), investigations of the relation to the D/E-rich domain of MBD3 are lacking. By identifying the DNA mimicry ability of the D/E-rich domain, we further demonstrated the interaction of the MBD and the D/E-rich domain contributing to dimer formation of the recombinant MBD3/MBDDE protein *in vitro*. As a result, the dimeric state restricts either the access of Zα to the D/E-rich domain or that of DNA to the MBD domain, leading to an autoinhibition state (Figures 4 and 5). Similar examples of autoinhibition have been defined in high mobility group protein B1 (HMGB1) and DNA methyltransferase 1 (Dnmt1). The acidic tail of HMGB1 is shielded by its basic HMG boxes, while the binding of HMG boxes to DNA releases the acidic tail from intramolecular interactions, allowing it to interact with the basic tail of histone H3 (48,49). The N-terminal replication focus targeting sequence (RFTS) domain of Dnmt1 inhibits the DNA-binding activity of Dnmt1, and the crystal structure of the RFTS domain suggests that the acidic loops of RFTS domain bind to the active site of the methyltransferase domain of Dnmt1, resulting in the exclusion of DNA (50).

Thus, autoinhibition serves as a regulatory mechanism to modulate the affinity of protein-protein or protein-DNA interactions and thereby controls their functions in response to changes in the environment. By using a photon counting histogram, Cui *et al.* analyzed the *in vivo* molecular stoichiometry of MBD3 and found that the extent of the homomeric state of MBD3 varies in the different cell phases (51). Based on a two-site sequencing binding mode in which the MBD domain sequentially dimerizes onto a single CpG site under physiological conditions (52), they proposed that MBD3 could adopt a homodimeric association with its epigenomic loci. Since our results show that MBD3 forms a homodimer independent of its DNA-binding ability, it is presumed that MBD3 protein can adopt a homodimeric association in cells and may switch between the DNA-free and DNA-bound states. The transition of two states is correlated with the binding of Zα to the D/E-rich domain.

### Zα and MBD3 regulate the transition of the DNA conformation

A conformational change in DNA impacts protein binding and is simultaneously influenced by protein binding. The Zα protein is a specific DNA-binding protein, which can induce and stabilize Z-DNA formation, while a B-DNA-binding protein, like the MBD domain, dose not exhibit activity (Figure 6A–C). Z-DNA forms in sequences composed of alternating purine and pyrimidine bases, such as CpG repeats. Cytosine methylation facilitates Z-DNA formation (5,53). The CpG repeats in the genome may modulate the thermodynamic propensity to form Z-DNA by affecting the dynamic properties of neighboring residues (54). Z-DNA is potentially formed near the promoter regions in actively transcribed genes because of the negative torsion strain induced by a moving polymerase or the chromatin remodeling complex (6,7,55–57). Furthermore, the association of Z-DNA formation with active transcription has been confirmed by ChIP-seq analysis (17). MBD3 displays a similar DNA sequence preference as Zα. Genome-wide mapping analyses have shown that MBD3 is localized in the CpG-rich promoter (24,58,59). The use of atomic level information provided by chemical shift analyses have shown that MBD3 preferentially localizes to methylated and, to a lesser degree, unmethylated CpG sites (39).

Accordingly, the Z-DNA structure formed in the promoter regions during transcription can be recognized and stabilized by Z-DNA binding proteins (18,60), which may be recruited by RNA polymerase or the chromatin remodeling complex as suggested by the pull-down assay (Figure 1C). The Z-DNA structure prevents nucleosome formation and maintains chromatin in an open state that is permissive for transcription factor binding, which is able to alter gene expression (7,8,14,18). For example, the Z-DNA-forming sequences in the CSF1 gene promoter are required for its activation by BRG1, a component of the BAF complex. BRG1 induces Z-DNA formation, and the Z-DNA structure inhibits nucleosome reformation. Although the MBD domain of MBD3 cannot compete with Zα for Z-DNA binding, the MBD domain may bind to the nearby regions close to the Z-DNA-forming sites. Binding of MBD3 to DNA releases the D/E-rich domain, which is able to substitute Z-DNA for Zα binding. Without being bound by Zα, the Z-DNA becomes relatively unstable and then relaxes back to B-DNA. Thus, the transition of the DNA conformation between B- and Z-DNA can be regulated by the interplay of MBD3 and Zα in a manner that is dependent on the context in cells. The conformational change in DNA impacts the chromatin accessibility and thereby modulates gene expression. Interestingly, a study of the MBD3/NuRD and BAF complex has suggested that the two complexes function in opposition to fine-tune gene expression (24). MBD3 associates with BRG1 *in vivo*, and MBD3 localization is lost upon knockdown of BRG1 in mouse embryonic stem cells (24,61,62). In addition, MBD3 and BRG1 play antagonistic roles in regulating the nucleosome occupancy and recruitment of RNA polymerase II at promoters of their target genes.

According to our findings, it is possible that for genes with Z-DNA-forming sites in promoters with opposite regulation driven by the MBD3/NuRD and BAF complex is correlated with the transition of DNA conformation mediated by the interplay between Z-DNA/Z-DNA binding proteins and the D/E-rich domain of MBD3.

### MBD3 interacts with ADAR1 *in vivo*

The interaction of MBD3 and ADAR1 in cells was first demonstrated in this study. Their biological function requires further investigation. Noticeably, both proteins are involved in the regulation of ESC pluripotency and differentiation. Mbd3-deficient mouse ESCs can maintain self-renewal without leukemia inhibitory factor but are unable to commit to the developmental lineage (21). Further, the Mbd3/NuRD complex directly regulates the expression of pluripotency genes (63). ADAR1-deficient human ESCs display profound defects in differentiation and neural induction (64). This phenotype is due in part to abnormal miRNA production after disruption of ADAR1. In contrast, RNA editing levels of Alu elements, a major target of ADAR1, are decreased in ADAR1 knockdown hESCs (65,66). Alu elements belong to the family of short interspersed elements and are the most abundant repetitive elements in the human genome (67). Alu RNA levels are increased in hESCs and their RNA editing levels are higher in undifferentiated compared with differentiated cells (68). Many studies have demonstrated that the change of Alu RNA and RNA editing levels can affect gene expression and genome integrity (67,69–72). Thus, it is possible that MBD3 and ADAR1 may regulate a set of common targets, genes or repetitive elements related to ESC pluripotency and differentiation. According to our findings, their expression levels may be finely tuned by regulation of the DNA conformational transition of promoter regions.

The IP results showing that the interaction of MBD3 and ADAR1 occurred not only through the interaction of the DE-rich domain and Zα may be due in part to the potential interactions between Zα and some components of the NuRD complex observed by the pull-down assays (Figure 1C). The components may assist MBD3 in associating with ADAR1. It is also possible that the interaction of MBD3 and ADAR1 may be through multiple regions of two proteins besides Zα and the D/E-rich domain. Since ADAR1-p150 is an interferon-inducible protein, the interaction of Zα and the D/E-rich domain may occur more predominantly under certain cellular conditions. The unexpected finding that ADAR1-p110, a constitutively expressed protein and lacking Zα, was also pulled down by FLAG-MBD3 indicating different functional properties when MBD3 interacts with different isoforms of ADAR1.

In conclusion, we present for the first time that a protein sequence with D/E-rich residues has the ability to compete with Z-DNA for Zα binding. In addition, our data suggest that the interplay between Z-DNA binding protein and MBD3 regulates the transition between B- and Z-DNA, resulting in alterations of gene expression. Certain questions that must be addressed in future studies are whether other proteins with D/E-rich residues possess the same properties and which Z-DNA-binding protein is involved in the regulatory machinery. For example, the interaction of ADAR1 and NF90 stimulates gene expression, while the ADAR1 mutant lacking the Z-DNA binding domain has lost its function (73). Furthermore, given that the amount of Z-DNA plays a role in disease development, e.g., systemic lupus erythematosus, autoimmune disease and Alzheimer's disease (74–76), the ability of D/E-rich peptide to mimic Z-DNA and to regulate Z-DNA content in cells may have valuable potential for therapeutic use.

## Supplementary Material

Supplementary Data
