# Supplementary material for: The C-terminal D/E-rich domain of MBD3 is a putative Z-DNA mimic that competes for Zα DNA-binding activity
Source: Nucleic Acids Res. 2018 Oct 10;46(22):11806–21. doi: 10.1093/nar/gky933 (PMC6294567; doi:10.1093/nar/gky933)
Supplement: Supplementary Data [file gky933_supplemental_files.pdf]

Supplementary data of
The C-terminal D/E-rich domain of MBD3 is a Z-DNA mimic that competes for Zα DNA-binding activity

Chi-Hua Lee, Yan-Ping Shih, Meng-Ru Ho, and Andrew H.-J. Wang*

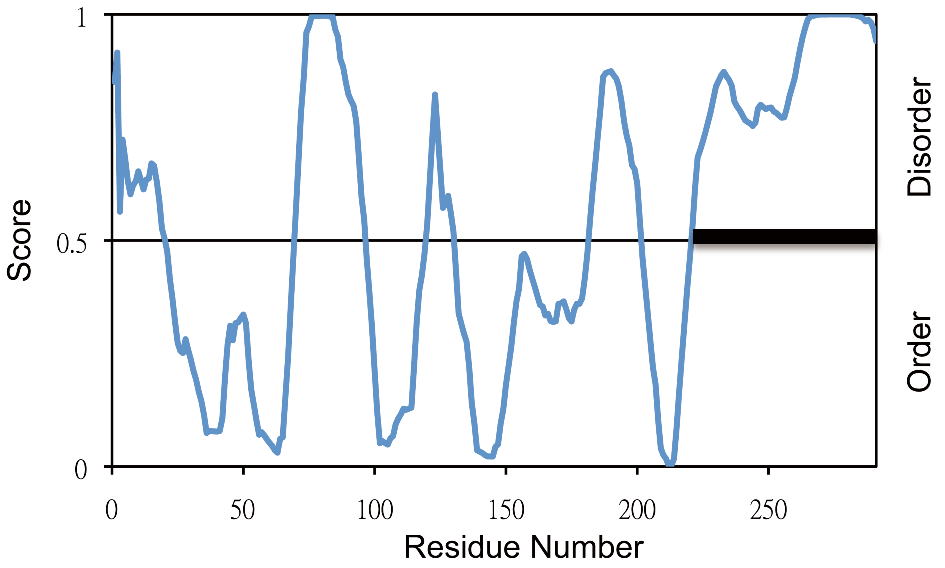

221 TDEDIRKQEE LVQQVRKRLE
EALMADMLAH VEELARDGEA
PLDKACAEDD DEEDEEEEEE
EPDPDPEMEH V 291

Figure S1. Scores of intrinsic disorder were predicted using PONER®VL-XT (http://www.pondr.com)(1)

REFERENCE

1. Romero, P., Obradovic, Z., Li, X., Garner, E.C., Brown, C.J. and Dunker, A.K. (2001) Sequence complexity of disordered protein. *Proteins*, **42**, 38-48.
